# CMTM5 influences Hippo/YAP axis to promote ferroptosis in glioma through regulating WWP2‐mediated LATS2 ubiquitination

**DOI:** 10.1002/kjm2.12889

**Published:** 2024-08-21

**Authors:** Ye Fan, He‐Qin Zou

**Affiliations:** ^1^ Brain Hospital of Hunan Province The Second People's Hospital of Hunan Province Changsha Hunan China

**Keywords:** CMTM5, ferroptosis, glioma, Hippo/YAP signaling, WWP2

## Abstract

Glioma, a common malignancy, is characterized by high morbidity and mortality. Promoting ferroptosis can delay tumor progression. Here, we aimed to explore the underlying mechanism of ferroptosis in glioma. In vitro and in vivo experiments were conducted using glioma cells and nude mice. The expression of genes and proteins was evaluated by RT‐qPCR, Western blot assay, and immunohistochemical staining. Malignant activities of glioma cells were evaluated using MTT, EdU, and Transwell assays. The levels of Fe^2+^, lipid reactive oxygen species, and malondialdehyde were determined using commercial kits. The interplays among CMTM5, WWP2, and LATS2 were validated using Co‐immunoprecipitation assay. The UALCAN database predicted downregulation of CMTM5 expression in glioma, and low expression of CMTM5 was associated with poor survival outcomes. CMTM5 overexpression inhibited cell growth and invasion and promoted ferroptosis of glioma cells. Besides, CMTM5 protein interacted with WWP2 protein and decreased WWP2 expression. WWP2 silencing attenuated LATS2 ubiquitination to enhance LATS2 expression and phosphorylation of YAP1. CMTM5 exerted a suppressive effect on cell growth and invasion and promoted ferroptosis of glioma cells by regulating the WWP2/LATS2 pathway. In the in vivo experiments, CMTM5 overexpression suppressed tumor growth and enhanced ferroptosis. CMTM5 regulated Hippo/YAP signaling to inhibit cell growth and invasion and to promote ferroptosis in glioma by regulating WWP2‐mediated LATS2 ubiquitination, thereby attenuating glioma progression.

## 1 INTRODUCTION

Glioma accounts for approximately 80% of all malignant tumors in the central nervous system.^1^ Owing to its invasive growth and a high propensity for recurrence, glioma is associated with a poor prognosis and high mortality.^2^ The standard clinical therapy for glioma includes maximum safe surgical resection combined with postoperative concurrent chemoradiotherapy.^3^ However, chemotherapeutic resistance and adverse reactions to chemoradiotherapy adversely affect the prognosis and quality of life of these patients.^4^ An accumulating body of evidence has shown that ferroptosis (an iron‐dependent programmed cell death that is different from apoptosis, cell necrosis, and autophagy) is closely related to various tumors, including glioma.^5^ Therefore, we sought to identify methods to diagnose and treat glioma from the perspective of ferroptosis.

CKLF like MARVEL transmembrane domain containing 5 (CMTM5) is a member of the chemotaxis‐like factor superfamily, which has leukocyte chemotaxis activity and also plays a key role in the blood system, immune system, reproductive system, and other systems.^6^ Several members of the CMTM family play a key role in the progression of malignant tumors.^7^ Available evidence suggests a suppressive effect of CMTM5 in various tumors.^8^ For instance, Peng Li and co‐workers suggested that lower CMTM5 expression contributed to the progression of epithelial ovarian cancer.^9^ Besides, CMTM5 was shown to be abnormally inhibited in prostate cancer and its overexpression was found to suppress the growth of prostate cancer cells.^10^ However, the potential role of CMTM5 in glioma and the underlying molecular mechanism is not well characterized.

WW domain containing E3 ubiquitin protein ligase 2 (WWP2) is an E3 ubiquitin protein ligase, belonging to the HECT type E3 ubiquitin ligase NEDD4‐like protein family.^11^ WWP2 has been extensively studied in several diseases. It has been found to regulate various cellular functions, such as cell proliferation, differentiation, apoptosis, and immune and inflammatory responses.^12^ WWP2 was identified as an accelerant for tumor progression.^13^ Yang et al. found that the reduced expression of WWP2 contributed to inhibiting lung adenocarcinoma cell migration and invasion.^14^ Additionally, Zou et al. proposed that WWP2 may promote gastric cancer by mediating the ubiquitination of large tumor suppressor kinase 1 (LATS1) protein and reducing the expression of LATS1.^15^ A few studies have investigated the role of WWP2 in glioma. Liang et al. demonstrated enhanced expression of WWP2 in glioma, which was related to its recurrence.^16^ Intriguingly, bioinformatics software predicted the interaction between WWP2 and CMTM5 proteins. However, the relationship between CMTM5 and WWP2 in glioma remains to be further elucidated.

The Hippo signaling pathway is a conserved signaling pathway in *Drosophila melanogaster*, mainly consisting of a series of protein kinases including hSAV1, MST1/2, large tumor suppressor kinase 1/2 (LATS1/2), MOB1A/B, TAOK1/3, etc. and transcriptional coactivators including YAP/TAZ and TEADs.^16^ Several studies have demonstrated abnormal expression of the main core components of the Hippo signaling pathway in tumor tissues.^17^ For example, low expression of LATS1 was reported in glioma and decreased LATS1 expression was associated with unfavorable prognosis.^18^ Another study revealed decreased expression of LATS2 in glioma tissues and cells, and LATS2 overexpression was found to suppress the growth of glioma cells.^19^ However, the underlying mechanism of low expression of LATS2 in glioma is not clear. Bioinformatics analysis identified LATS2 as a potential substrate of WWP2 E3 ubiquitin ligase. Therefore, we speculated that WWP2 might regulate Hippo/YAP signaling axis‐mediated glioma progression by mediating LATS2 ubiquitination.

Based on the above‐mentioned findings, we hypothesized that CMTM5 interacts with WWP2 to influence WWP2‐mediated LATS2 ubiquitination, which further influences glioma progression by mediating glioma cell growth and ferroptosis through regulation of the Hippo/YAP axis. We believe that our findings may help identify more targeted genes for glioma therapy.

## 2 MATERIALS AND METHODS

### 2.1 The University of Alabama at Birmingham Cancer Data Analysis Portal (UALCAN) database

The UALCAN database was used to examine the expression of CMTM5 and the relationship between CMTM5 expression and survival outcomes in glioma. The UALCAN database is a comprehensive, user‐friendly, and interactive web resource for analyzing cancer omics data. The database provides a graphical representation of gene expression and the relationship between gene expression and survival outcomes.^20^

### 2.2 Cell culture and treatment

Glioma cell lines A172 and LN229 (ATCC, VA, USA) were maintained in RPMI 1640 medium (Thermo Fisher Scientific, Massachusetts, USA), containing 10% FBS (Thermo Fisher Scientific), 100 μg/mL streptomycin, and penicillin (Beyotime, Shanghai, China), in a saturated humidity incubator at 37°C and 5% CO_2_.

For erastin treatment, 10 μM erastin (Selleck Chemicals, Texas, USA) was added to glioma cells for 48 h incubation. For ferrostatin‐1 (Fer‐1) treatment, 1 μM Fer‐1 (MedChem Express, New Jersey, USA) was added to glioma cells for 48 h incubation.

### 2.3 Cell transfection

Short hairpin targeting CMTM5 (sh‐CMTM5) or WWP2 (sh‐WWP2) or LATS2 (sh‐LATS2) along with the corresponding controls (sh‐NC) were purchased from GenePharma (Shanghai, China). Glioma cells were implanted onto 6‐well plates and incubated overnight. Subsequently, the cells were transfected with plasmids for 48 h using Lipofectamine™ 3000 (Invitrogen, CA, USA) following the manufacturer's instructions. In addition, glioma cells were transfected with lentivirus carrying oe‐CMTM5 GenePharma (China). The procedure for obtaining the lentivirus was as follows: HEK293T cells were used for lentivirus packaging. oe‐CMTM5 or pCDH‐DsRed was co‐transfected with lentivirus packaging plasmids psPAX2 and pMD2G using LipoFiter 3.0 when cell density had reached approximately 50%. Six hours later, fresh medium was added after the removal of the supernatant. After 48 h of cultivation, virus supernatants were obtained and then were filtered with a 0.45 μm filter membrane. Lentivirus transfection into glioma cells was performed using virus supernatants and polybrene (8 μg/mL). Subsequently, puromycin was used to select infected glioma cells after 72 h.

### 2.4 RNA isolation and quantitative real‐time polymerase chain reaction (RT‐qPCR)

The relative expression of genes was determined by RT‐qPCR. Briefly, using TRIzol reagent (Beyotime), total RNA was acquired from glioma cells. The extracted RNAs were used for cDNA synthesis using Script Reverse Transcription Reagent Kit (TaKaRa, Osaka, Japan). qPCR process was performed using the SYBR Premix Ex Taq II Kit (TaKaRa). The relative expression of targeted genes was computed using the 2^−ΔΔCt^ formula. GAPDH acted as a reference gene. The primer sequences are presented in Table 1.

**TABLE 1:** Primers used in the study.

	Forward primer (5′–3′)	Reverse primer (5′–3′)
CMTM5	CTTCCTCACCTCCCACAAG	AGATGGAAACCAGGATGATG
WWP2	AGGCTAAAGAGGGCTGGAGT	GCTTTGCGGACACCACTTTC
LATS2	ACAAGATGGGCTTCATCCAC	CTCCATGCTGTCCTGTCTGA
GAPDH	CTGACTTCAACAGCGACACC	GTGGTCCAGGGGTCTTACTC

### 2.5 Western blot assay

Total proteins were extracted from glioma cells and tumor tissues harvested from nude mice using RIPA lysis buffer (Beyotime). The concentration of extracted proteins was determined using the BCA method. Afterward, equal proteins were loaded in each well to segregate the proteins using 10% sodium dodecyl sulfate‐polyacrylamide gel electrophoresis (SDS‐PAGE). Subsequently, the separated proteins were transferred onto polyvinylidene fluoride (PVDF) membranes. After blocking with skimmed milk (5%), the PVDF membranes were incubated overnight with the following primary antibodies at 4°C: CMTM5 (PA5‐50500, 1:2000), WWP2 (ab103527, 1:5000), LATS2 (PA5‐120433, 1:2000), YAP1 (PA1‐46189, 1:1000), p‐YAP1 (PA5‐17481, 1:1000), GPX4 (ab125066, 1:5000), SLC7A11 (ab307601, 1:1000), and GAPDH (ab9485, 1:2500). CMTM5, LATS2, YAP1, and p‐YAP1 antibodies were purchased from Thermo Fisher Scientific while the other antibodies were obtained from Abcam (Cambridge, UK). Next, the membranes were incubated with HRP‐conjugated secondary antibody (A0208, 1:1000, Beyotime) for 1 h. The protein bands were visualized using the ECL kit (Beyotime). ImageJ was used for the densitometry analysis of gray values.

### 2.6 3‐(4,5‐Dimethylthiazol‐2‐yl)‐2,5‐diphenyltetrazolium bromide (MTT) assay

Glioma cells (2 × 10^4^ cells/well) were planted in a 96‐well plate and cultured overnight. Then, MTT (Sigma‐Aldrich, Missouri, USA) was added for 1 h. The absorbance at 570 nm was determined by a Tecan Infinite M200 reader (Switzerland).

### 2.7 5‐Ethynyl‐2′‐deoxyuridine (EdU) assay

Glioma cells were seeded into 6‐well plates and cultured overnight. Afterward, 10 μM EdU was added for 2 h. Paraformaldehyde (4%) was used for the fixation of cells and 0.3% Triton X‐100 was applied to permeate cells. Then, a click reaction solution (Beyotime) was added and the cells were incubated. Additionally, cells were stained using DAPI. Images were captured within 24 h using an inverted fluorescent microscope (Olympus, Tokyo, Japan).

### 2.8 Transwell assay

A 24‐well Transwell insert system (Corning, New York, USA) was applied in this experiment. To detect cell migration, glioma cells that had been intervened were implanted in the upper chamber containing FBS‐free RPMI 1640 medium with 1% mitogenase. RPMI 1640 medium supplemented with 10% FBS was added to the lower chamber. Cells migrating to the lower chamber after 24 h were fixed with 95% alcohol, stained with 1% crystal violet (Sigma‐Aldrich), and examined using an inverted microscope (Olympus).

For cell invasion assay, except for the top Transwell chamber filled with Matrigel (Becton, New Jersey, USA), the remaining processes were the same as those used for detecting cell migration.

### 2.9 The evaluation of Fe^2+^, lipid reactive oxygen species (ROS), and malondialdehyde (MDA) levels

The MDA content in glioma cells was quantified using an MDA assay kit (Beyotime), following the manufacturer's instructions. The ROS content in glioma cells was determined using a ROS detection kit (Beyotime). The Fe^2+^ level in glioma cells was measured using an iron assay kit (Solarbio, Beijing, China).

### 2.10 Stability of LATS2 after CHX treatment and LATS2 expression of MG132 treatment

Glioma cells transfected with sh‐NC or sh‐WWP2 were treated with 12.5 μg/mL cycloheximide (CHX) (MedChemExpress) for 0, 2, 4, 6, and 8 h, respectively. The protein levels of LATS2 were measured using Western blot.

A proteasome inhibitor MG132 (10 μg/mL, MedChemExpress) was added to glioma cells for 4 h. Subsequently, the alteration of WWP2 and LATS2 protein levels was detected using Western blot.

### 2.11 Co‐immunoprecipitation (Co‐IP) assay

Glioma cells were lysed with Co‐IP buffer. Then, the lysates were incubated overnight with antibodies including IgG or CMTM5 (c‐374206, Santa Cruz, CA, USA) or WWP2 (ab103527, Abcam) or LATS2 (ab243657, Abcam) antibodies conjugated to Protein A/G beads (Santa Cruz). After beads were washed, immune‐precipitates including WWP2, ubiquitin, and LATS2 were detected by Western blot.

### 2.12 Tumor formation in nude mice

Nude mice (male, 3–5 weeks of age) were randomly divided into four equal groups (*n* = 5 for each group). Mice were purchased from Brain Hospital of Hunan Province, The Second People's Hospital of Hunan Province. A172 cells (2 × 10^6^ cells) infected with lentivirus carrying oe‐CMTM5 were inoculated into the subcutaneous axilla of the left forelimb of nude mice. Tumor volume was assessed every 7 days until day 35. Then, mice were euthanized and tumor tissues were completely harvested for immunohistochemistry (IHC) and Western blot assay. Animal experiments were approved by the ethics committee of Brain Hospital of Hunan Province, The Second People's Hospital of Hunan Province.

### 2.13 Immunohistochemistry (IHC)

Tumor tissues from nude mice were fixed with 4% paraformaldehyde and paraffin‐embedded. After repairing the antigen, the sections were blocked with 1% BSA. Subsequently, the sections were stained with antibodies against Ki67 (ab15580, Abcam) and GPX4 (ab125066, Abcam). The sections were then treated with HRP‐labeled antibodies and counterstained with diaminobenzidine (DAB, Beyotime). The images were acquired using an Olympus microscope (Japan).

### 2.14 Statistical analysis

The data were presented as mean ± standard deviation (SD). GraphPad Prism 8.0 was used for data analysis. Student's *t*‐test was used to compare continuous variables between two groups and analysis of variance (ANOVA) was used for multi‐group comparisons. All data were derived from three independent experiments. *p* < 0.05 was considered indicative of statistical significance.

## 3 RESULTS

### 3.1 CMTM5 overexpression promoted erastin‐induced ferroptosis of glioma cells and inhibited malignant characteristics of glioma cells

**FIGURE 1:**
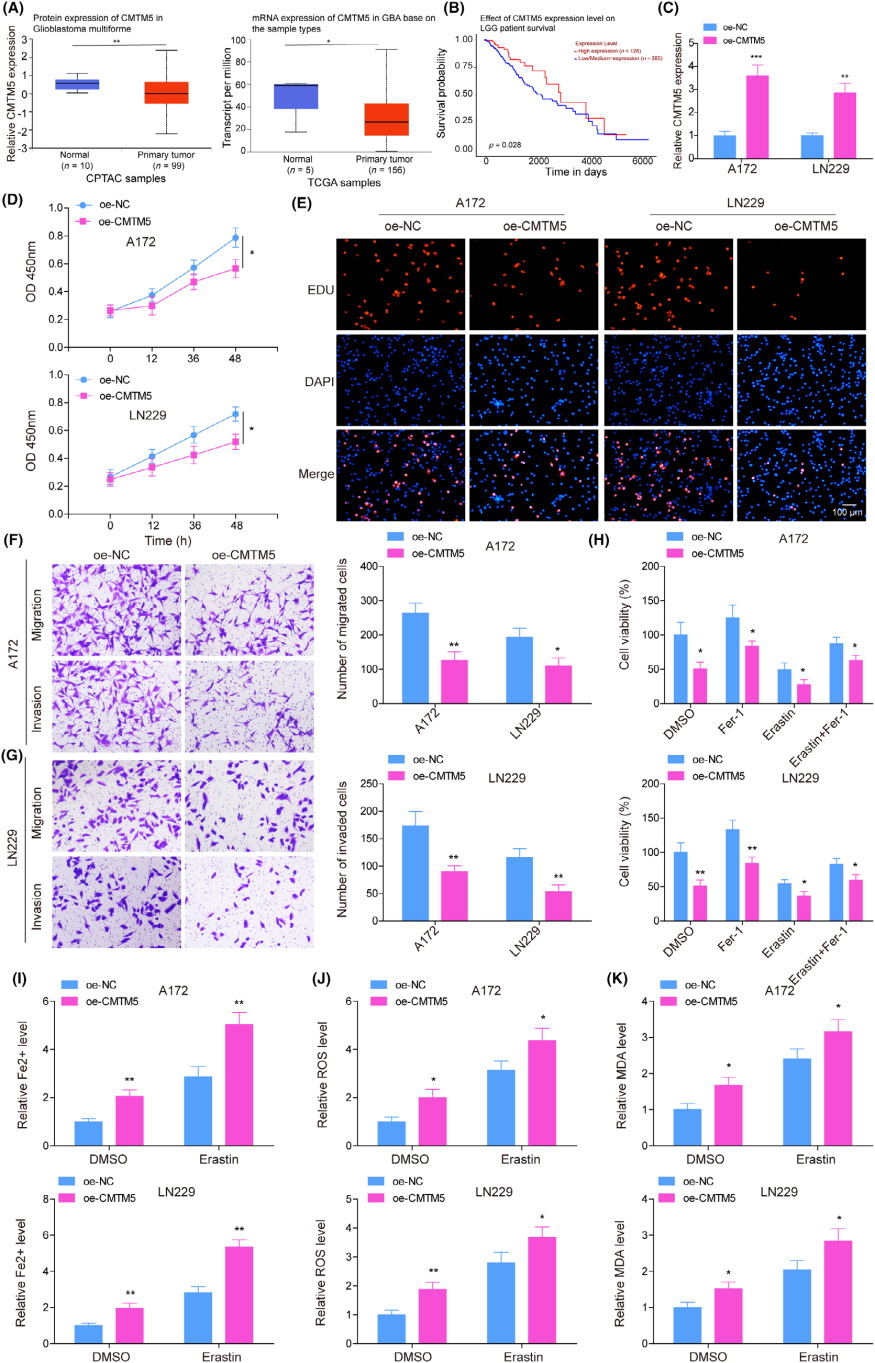
CMTM5 overexpression promoted erastin‐induced ferroptosis of glioma cells and inhibited malignant characteristics of glioma cells. (A, B) CMTM5 expression in glioblastoma multiforme and the relationship between CMTM5 expression and survival outcomes in LGG (brain lower grade glioma) as predicted by the UALCAN database. Glioma cells were transfected with oe‐NC or oe‐CMTM5. (C) CMTM5 expression was detected using RT‐qPCR. (D) Cell viability was evaluated using an MTT assay. (E) Cell proliferation was quantified by EdU assay. (F, G) Cell migration and invasion were determined using Transwell assay. Fer‐1 or/and erastin was/were added to glioma cells with/without oe‐CMTM5 transfection. (H) Cell viability was evaluated using an MTT assay. Glioma cells with/without oe‐CMTM5 transfection were added with/without erastin. (I–K) The levels of Fe^2+^, lipid ROS, and MDA were determined using commercial kits. *p < 0.05, **p < 0.01, ***p < 0.001.

Previous studies have identified CMTM5 as a tumor suppressor in several cancers.^8^, ^9^ First, predictive analysis using the UALCAN database showed lower expression of CMTM5 in glioblastoma multiforme. In addition, low CMTM5 expression in lower‐grade glioma (LGG) was associated with poor survival (Figure 1A,B). In addition, we further detected the expression in different grades of glioma and found that increased CMTM5 in lower‐grade glioma (Figure S1A). Meanwhile, there was no difference in the expression of CMTM5 in the mutant and non‐mutated groups compared with the normal group (Figure S1B). To investigate the mechanism by which CMTM5 influences the growth of glioma cells, two cell lines of glioma cells (A172 and LN229 cells) were transfected with oe‐CMTM5 to overexpress CMTM5. As presented in Figure 1C, RT‐qPCR indicated enhanced CMTM5 expression in glioma cells transfected with oe‐CMTM5. Subsequently, CMTM5 overexpression was found to suppress cell viability and cell proliferation (Figure 1D,E). Transwell assay showed that CMTM5 overexpression significantly attenuated cell migration and invasion of A172 and LN229 cells (Figure 1F,G). As previously described, ferroptosis is closely related to tumors.^21^ CGGA database (http://www.cgga.org.cn/index.jsp) analysis showed that CMTM5 was negatively correlated with the ferroptosis‐related protein GPX4, suggesting that CMTM5 might promote ferroptosis in glioma (Figure S2A). Therefore, Fer‐1 (ferroptosis inhibitor) or/and erastin (ferroptosis inducer) was/were added to A172 and LN229 cells with/without oe‐CMTM5 transfection. Fer‐1 was promoted while erastin inhibited the viability of A172 and LN229 cells. Of note, the viability of oe‐CMTM5‐transfected A172 and LN229 cells was inhibited when compared to the corresponding control groups (Figure 1H). Furthermore, to probe the impact of CMTM5 on ferroptosis in glioma cells, glioma cells with/without oe‐CMTM5 transfection were added with/without erastin. As shown in Figure 1I–K, overexpression of CMTM5 increased the levels of Fe^2+^, lipid ROS and MDA. The indicators of ferroptosis including Fe^2+^, lipid ROS, and MDA were enhanced by erastin treatment, which was further elevated by CMTM5 overexpression. Collectively, these findings indicate that CMTM5 overexpression promoted erastin‐induced ferroptosis and inhibited cell growth of glioma cells.

### 3.2 CMTM5 overexpression inhibited WWP2 expression through interacting with WWP2

**FIGURE 2:**
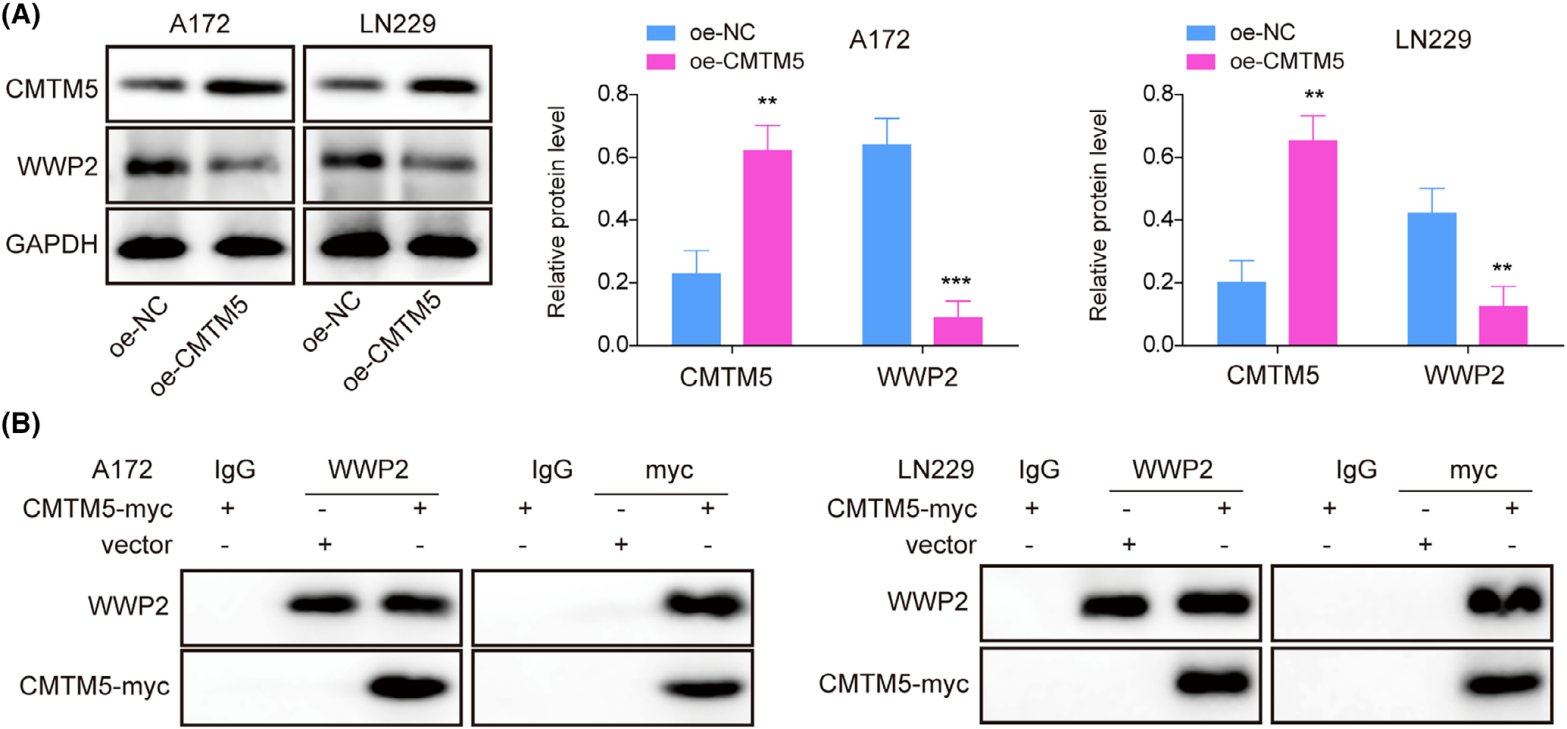
CMTM5 overexpression inhibited WWP2 expression by interacting with WWP2. (A) CMTM5 and WWP2 levels were detected in glioma cells transfected with oe‐NC or oe‐CMTM5 using Western blot. (B) The interaction between CMTM5 and WWP2 in glioma cells was verified using a co‐immunoprecipitation assay. *p < 0.05, **p < 0.01, ***p < 0.001.

As previously described, WWP2 was shown to have a promotive effect in tumors including glioma.^13^, ^16^ In oe‐CMTM5‐transfected A172 and LN229 cells, oe‐CMTM5 transfection upregulated CMTM5 expression while resulting in a decrease of WWP2 expression (Figure 2A). Moreover, Co‐IP analysis validated the interaction between CMTM5 and WWP2 in glioma cells (Figure 2B). These results suggested that CMTM5 may negatively regulate WWP2 expression through interacting with each other.

### 3.3 CMTM5 inhibited cell growth and promoted ferroptosis of glioma cells by inhibiting WWP2 expression

**FIGURE 3:**
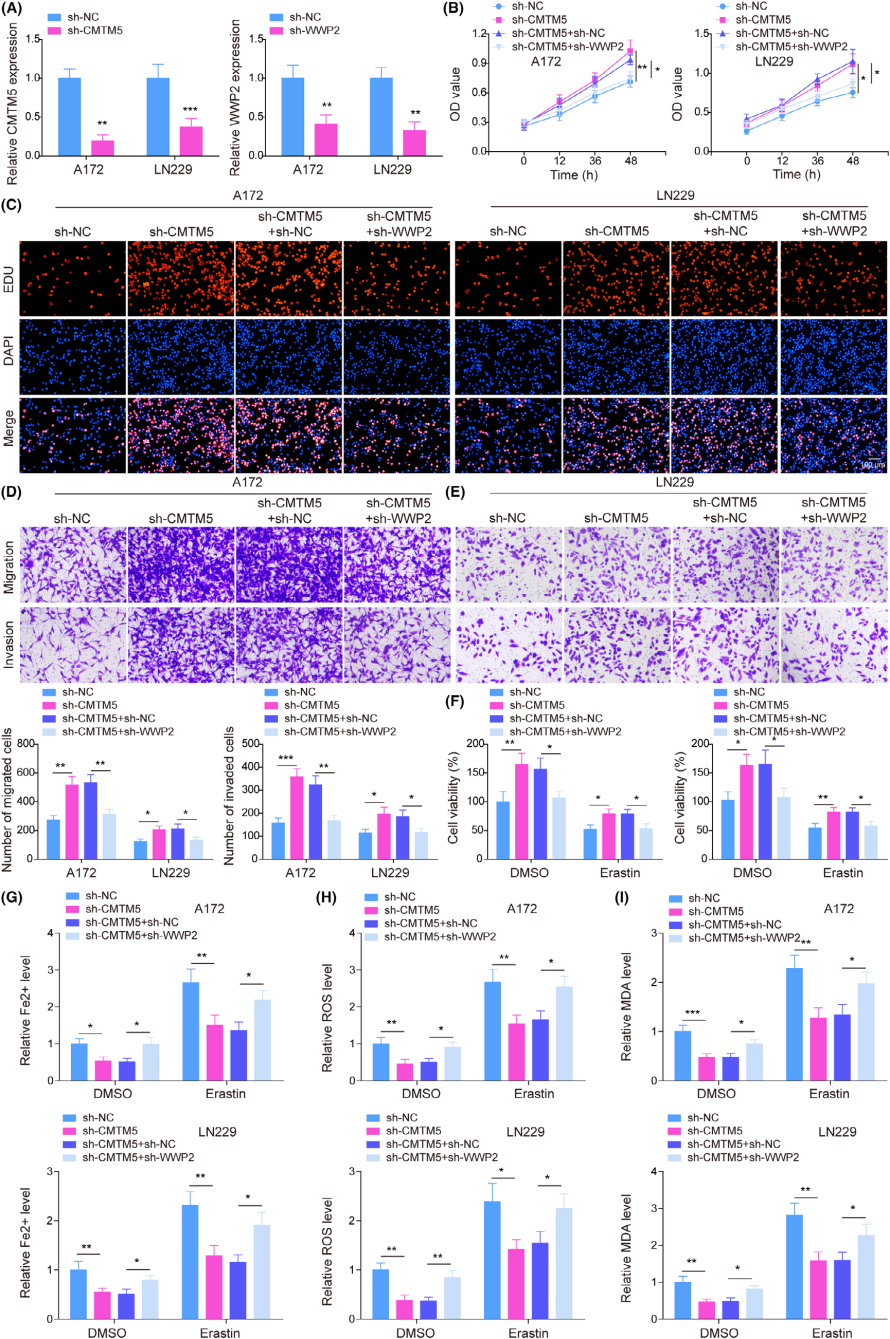
CMTM5 inhibited cell growth and promoted ferroptosis of glioma cells by inhibiting WWP2 expression. (A) CMTM5 and WWP2 expression was detected in glioma cells transfected with sh‐CMTM5 or sh‐WWP2 using RT‐qPCR. Glioma cells were transfected with sh‐CMTM5 or in combination with sh‐WWP2. (B) Cell viability was evaluated using MTT assay. (C) Cell proliferation was quantified by EdU assay. (D, E) Cell migration and invasion were determined using Transwell assay. Glioma cells were transfected with sh‐CMTM5 or in combination with sh‐WWP2 and followed by erastin treatment. (F) Cell viability was evaluated using MTT assay. Glioma cells with/without oe‐CMTM5 transfection were added with/without erastin. (G–I) The levels of Fe^2+^, lipid reactive oxygen species (ROS), and malondialdehyde (MDA) were determined using commercial kits. *p < 0.05, **p < 0.01, ***p < 0.001.

To explore the influence of the CMTM5/WWP2 axis on cell growth, A172 and LN229 cells received sh‐CMTM5 or in combination with sh‐WWP2 transfection. Figure 3A demonstrates the successful knockdown of CMTM5 and WWP2 in glioma cells with sh‐CMTM5 or sh‐WWP2 transfection. As shown in Figure 3B–E, CMTM5 knockdown led to increased cell viability, proliferation, migration, and invasion of glioma cells, while WWP2 silencing compromised these alterations caused by CMTM5 knockdown. Subsequently, the above‐treated cells were treated with erastin to investigate the effect of the CMTM5/WWP2 axis on ferroptosis. Knockdown of WWP2 reversed the promotion of CMTM5 knockdown on glioma cell viability. Erastin was found to reduce cell viability, while CMTM5 knockdown improved cell viability in erastin‐induced glioma cells, whereas WWP2 downregulation impaired these changes (Figure 3F). Additionally, CMTM5 knockdown reduced the level of Fe^2+^, lipid ROS, and MDA, while knockdown of WWP2 reversed the effect of CMTM5 shRNA on ferroptosis indicators. Erastin treatment enhanced Fe^2+^, lipid ROS, and MDA levels, which was offset by CMTM5 knockdown. However, knockdown of WWP2 rescued erastin‐induced ferroptotic cell death inhibited by CMTM5 deficiency (Figure 3G–I). Collectively, these findings suggest that CMTM5 knockdown augmented cell growth and suppressed ferroptosis of glioma cells by regulating WWP2.

### 3.4 CMTM5 regulated LATS2 ubiquitination by inhibiting WWP2 expression

**FIGURE 4:**
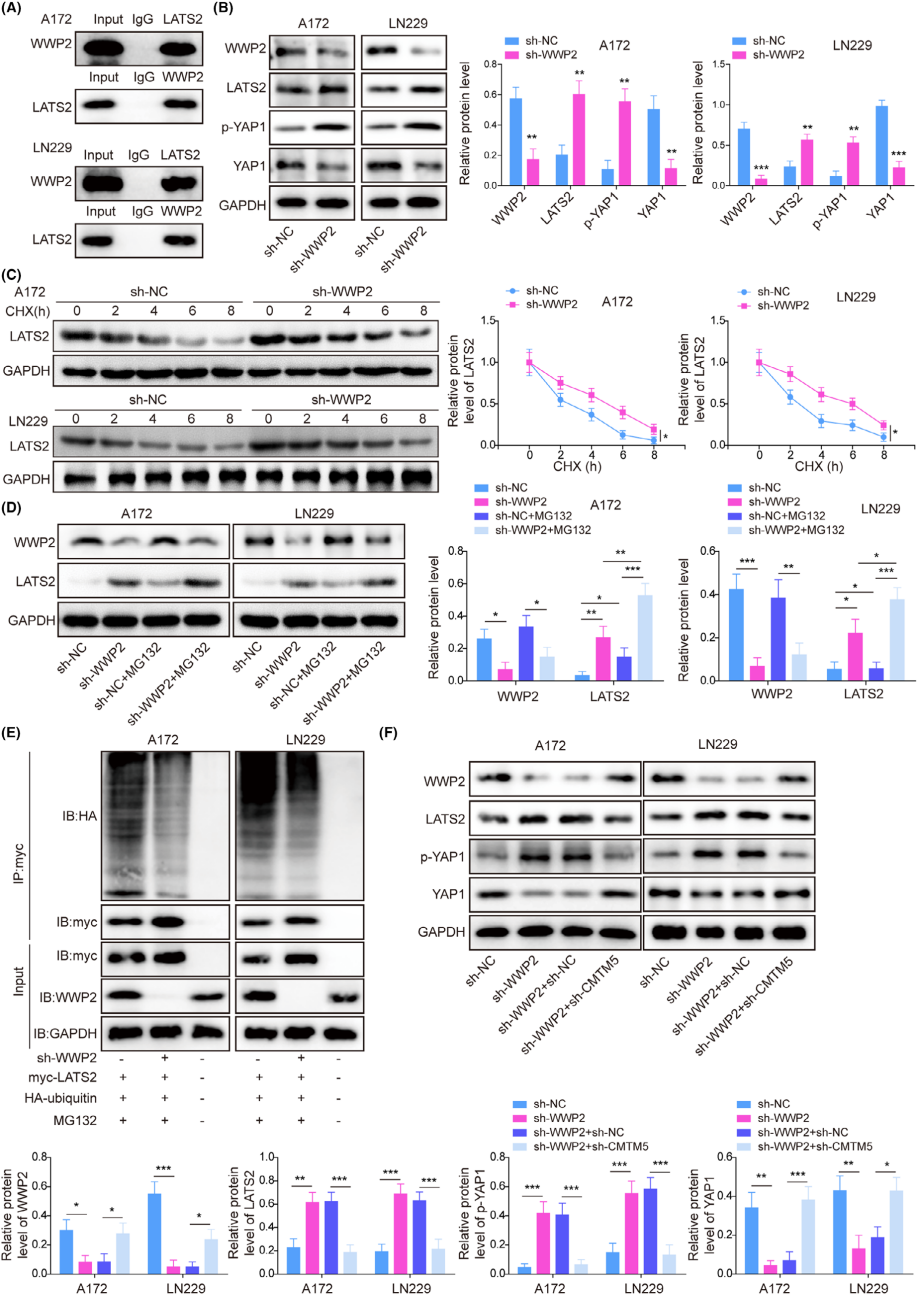
CMTM5 regulated LATS2 ubiquitination by inhibiting WWP2 expression. (A) The interaction between WWP2 and LATS2 in glioma cells was verified using a co‐immunoprecipitation (Co‐IP) assay. Glioma cells were transfected with sh‐WWP2. (B) WWP2, YAP1, LATS2, and p‐YAP1 levels were detected using Western blot assay. (C) The stability of LATS2 was determined using Western blot after CHX treatment. MG132 was added to glioma cells with/without sh‐WWP2 transfection. (D) WWP2 and LATS2 levels were evaluated using Western blot assay. (E) Ubiquitination of LATS2 was examined by Co‐IP. Glioma cells were transfected with sh‐WWP2 or together with sh‐CMTM5. (F) WWP2, YAP1, LATS2, and p‐YAP1 levels were detected using Western blot assay. *p < 0.05, **p < 0.01, ***p < 0.001.

LATS2, a core component of the Hippo/YAP axis, has been implicated in various diseases including cancers.^22^ Through Co‐IP assay, we observed the interaction between WWP2 and LAST2 (Figure 4A). Besides, WWP2 knockdown decreased WWP2 and YAP1 expression, while it increased LATS2 and p‐YAP1 levels in A172 and LN229 cells (Figure 4B). Further, WWP2 knockdown was found to enhance the stability of LATS2 protein in glioma cells (Figure 4C), indicating the potential effect of WWP2 on the ubiquitination of LATS2 protein. Thus, MG132, a proteasome inhibitor, was added to glioma cells with/without sh‐WWP2 transfection. MG132 treatment was found to further promote WWP2 knockdown‐mediated elevation of LATS2 expression in A172 and LN229 cells (Figure 4D). In addition, WWP2 knockdown significantly suppressed the ubiquitination of LATS2 (Figure 4E). As shown in Figure 2, WWP2 was negatively regulated by CMTM5. Furthermore, the CGGA database found that CMTM5 is negatively correlated with YAP1 (Figure S2B). Here, we found that WWP2 knockdown‐mediated reduction of WWP2 and YAP1 expression and elevation of LATS2 and p‐YAP1 levels in glioma cells was reversed by the combination of sh‐WWP2 and sh‐CMTM5 transfection (Figure 4F). Collectively, these findings indicate that CMTM5 inhibited WWP2‐mediated LATS2 ubiquitination in glioma cells.

### 3.5 CMTM5/WWP2 axis enhanced LATS2 expression to suppress cell growth and promote ferroptosis in glioma cells

**FIGURE 5:**
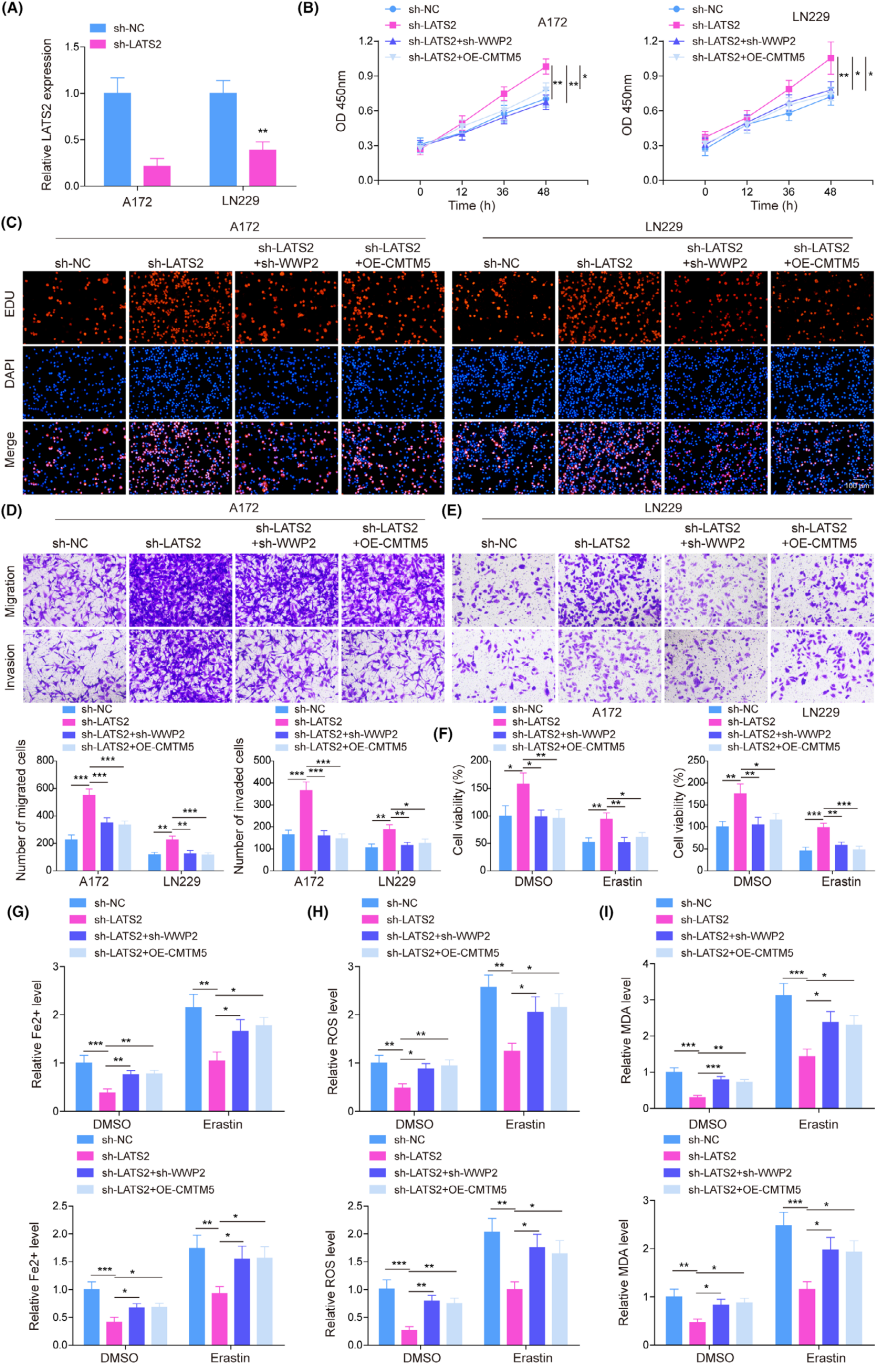
CMTM5/WWP2 axis enhanced LATS2 expression to suppress cell growth and promote ferroptosis in glioma cells. (A) LATS2 expression was detected in glioma cells transfected with sh‐LATS2 using RT‐qPCR. Glioma cells were transfected with sh‐LATS2 or together with sh‐WWP2/oe‐CMTM5 transfection. (B) Cell viability was evaluated using an MTT assay. (C) Cell proliferation was quantified by EdU assay. (D, E) Cell migration and invasion were determined using Transwell assay. Glioma cells were transfected with sh‐LATS2 or together with sh‐WWP2/oe‐CMTM5 transfection and followed by erastin treatment. (F) Cell viability was evaluated using an MTT assay. Glioma cells with/without oe‐CMTM5 transfection were added with/without erastin. (G–I) The levels of Fe^2+^, lipid reactive oxygen species (ROS), and malondialdehyde (MDA) were determined using commercial kits. *p < 0.05, **p < 0.01, ***p < 0.001.

A172 and LN229 cells were treated with sh‐LATS2 or together with sh‐WWP2/oe‐CMTM5 transfection. Figure 5A demonstrates a successful reduction in LATS2 expression in sh‐LATS2‐transfected cells. Besides, LATS2 downregulation promoted cell viability, proliferation, migration, and invasion of glioma cells, whereas WWP2 knockdown or CMTM5 overexpression reversed these changes caused by LATS2 downregulation (Figure 5B–E). Then, the above‐treated cells were subjected to erastin intervention. LATS2 knockdown promoted glioma cell viability, Erastin treatment suppressed cell viability and the variation tendencies of cell viability by sh‐LATS2/sh‐WWP2/oe‐CMTM5 transfection in erastin‐induced glioma cells mimicked those in glioma cells without erastin treatment (Figure 5F). Moreover, LATS2 knockdown reduced the level of Fe^2+^, lipid ROS, and MDA, while WWP2 knockdown or CMTM5 overexpression reversed the effect of sh‐LATS2 on ferroptosis indicators. Erastin treatment promoted levels of Fe^2+^, lipid ROS, and MDA in glioma cells, which was abolished by LATS2 knockdown. However, LATS2 knockdown‐mediated reduction of Fe^2+^, lipid ROS, and MDA levels in erastin‐induced glioma cells was reversed by WWP2 knockdown or CMTM5 overexpression (Figure 5G–I). These findings suggest that CMTM5 overexpression or WWP2 knockdown compromised LATS2 knockdown‐induced cell growth promotion and ferroptosis suppression in glioma cells.

### 3.6 Overexpression of CMTM5 inhibited glioma growth and promoted ferroptosis in vivo

**FIGURE 6:**
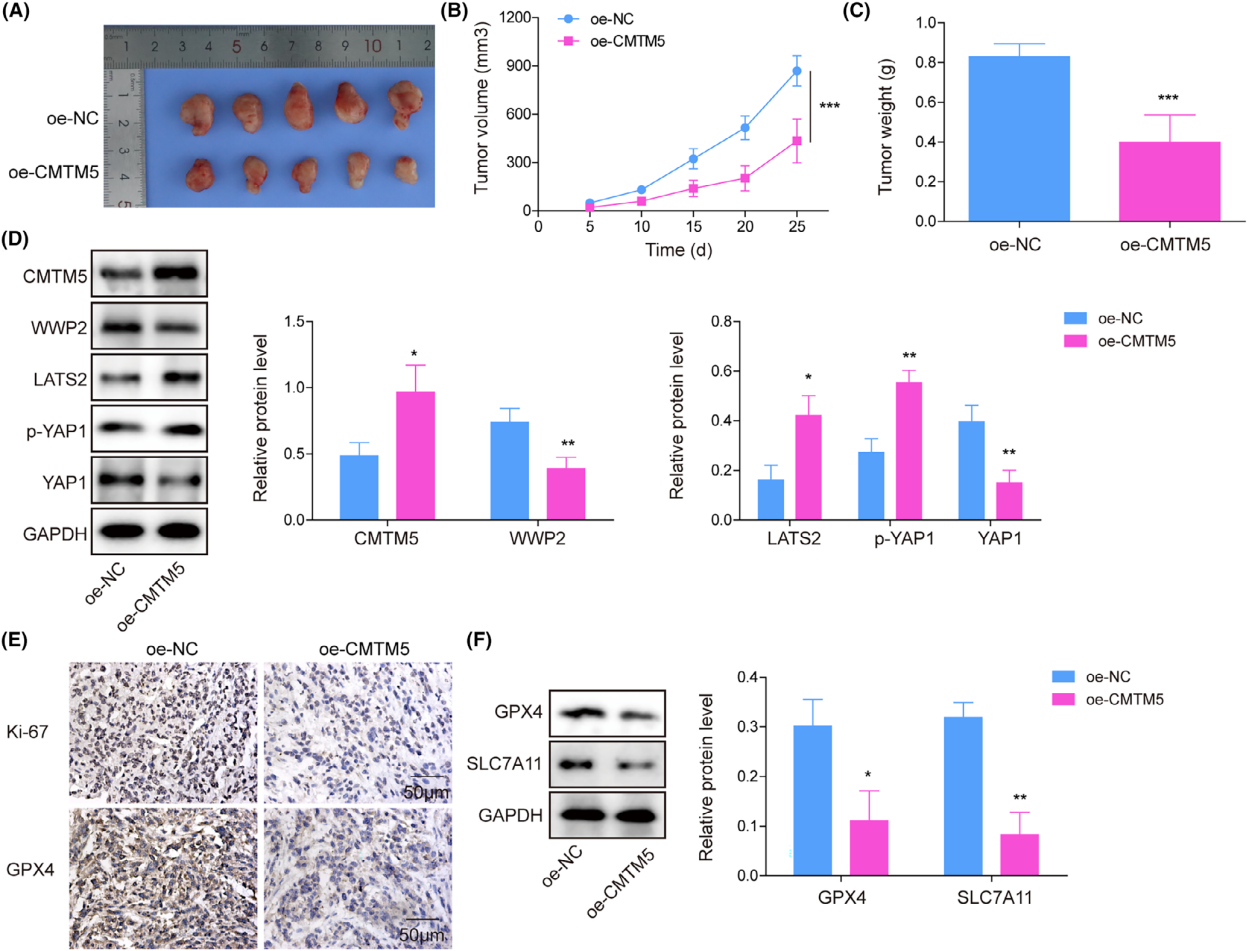
Overexpression of CMTM5 inhibited glioma growth and promoted ferroptosis in vivo. Nude mice were administered a subcutaneous injection of oe‐NC or oe‐CMTM5‐transfected A172 cells. (A) The tumor images; (B) the growth curve of tumor volume; (C) the weight of tumors. (D) CMTM5, WWP2, YAP1, LATS2, and p‐YAP1 levels were detected using Western blot assay. (E) Ki67 and GPX4 levels were evaluated using immunohistochemical staining. (F) GPX4 and SLC7A11 levels were measured by Western blot assay. *p < 0.05, **p < 0.01, ***p < 0.001.

To verify the effects of CMTM5 on glioma cells in vivo, nude mice were inoculated with A172 cells with oe‐NC or oe‐CMTM5 transfection. As indicated in Figure 6A–C, CMTM5 overexpression inhibited tumor volume and weight in nude mice. Additionally, CMTM5 overexpression elevated CTMT5, LATS2, and p‐YAP1 expression while it reduced WWP2 and YAP1 expression in tumors (Figure 6D). Besides, CMTM5 overexpression inhibited Ki‐67 (a marker of cell proliferation) and GPX4 (a marker of ferroptosis), as determined by IHC (Figure 6E). Furthermore, Western blot revealed that CMTM5 overexpression also decreased GPX4 and SLC7A11 (a marker of ferroptosis) expression (Figure 6F). These findings indicated that CMTM5 overexpression suppressed tumor growth and promoted ferroptosis in nude mice.

## 4 DISCUSSION

Glioma is an aggressive tumor with a high incidence and mortality rate. Moreover, it often occurs in children and adolescents, imposing a high morbidity and mortality burden.^23^ Promoting ferroptosis in tumor cells has been shown to significantly delay tumor progression.^24^ For instance, dihydroartemisinin was shown to promote cell ferroptosis to suppress glioma progression.^25^ This is the first study to demonstrate that CMTM5, which suppressed WWP2 expression through interacting with WWP2 protein, inhibited cell growth and promoted ferroptosis by attenuating WWP2‐mediated LATS2 ubiquitination and degradation, thereby alleviating glioma progression.

*CMTM5* gene encodes a chemokine superfamily member, encoding a protein with tumor suppressor effects.^26^ CMTM5 expression was decreased and attenuated malignant characteristics of hepatocellular carcinoma via the PI3K‐AKT pathway.^8^ CMTM5 also suppresses prostate cancer by inactivating the EGFR/PI3K/AKT pathway.^27^ These findings suggest that CMTM5 may also suppress glioma progression. In the present study, online database predicted reduced expression of CMTM5 in glioma. Furthermore, CMTM5 overexpression inhibited the growth and invasion and promoted ferroptosis of glioma cells, all of which indicated that CMTM5 could delay glioma progression.

However, the molecular mechanism underlying the effect of CMTM5 in glioma is not clear. Here, we analyzed the interplay between CMTM5 protein and WWP2 protein, which was validated using Co‐IP. Furthermore, we found that CMTM5 overexpression reduced WWP2 expression, indicating a negative relationship between CMTM5 and WWP2. WWP2 is a well‐known E3 ubiquitination ligase and several studies have demonstrated aberrant expression of E3 ubiquitination ligases in a variety of diseases, especially cancers.^28^, ^29^, ^30^ WWP2 has been implicated in several cancers, including glioma.^16^ For instance, WWP2 knockdown was found to suppress malignant activities by regulating the AKT pathway and augment the antitumor effect of doxorubicin in hepatocellular carcinoma.^31^ Besides, WWP2 silencing enhanced PTEN expression, reducing the growth of gastric cancer cells.^32^ More importantly, a previous study reported the suppressive effect of WWP2 silencing in glioma.^16^ The present study provides more evidence of the inhibitory effect of WWP2 knockdown in glioma and the related mechanism. Knockdown of WWP2 abolished CMTM5 silencing‐promoted cell growth and invasion and CMTM5 silencing‐suppressed ferroptosis of glioma cells. Collectively, this is the first study to demonstrate that CMTM5 overexpression may suppress cell growth and invasion and promote ferroptosis of glioma cells by reducing WWP2 expression.

As an important E3 ubiquitin protein ligase, WWP2 influences the levels of targeted substrate proteins by mediating substrate ubiquitination, thereby mediating the multi‐biological activities of cells.^15^, ^33^ PTEN and LATS1 have been demonstrated as substrate proteins of WWP2.^15^, ^33^ Here, we identified LATS2 as a substrate protein of WWP2 in glioma. Our experimental results displayed an interaction between WWP2 and LATS2 and WWP2 silencing resulted in increased LATS2 expression and reduced ubiquitination of LATS2. Of note, CMTM5 silencing reversed WWP2 silencing‐mediated enhancement of LATS2 expression. LATS2 is a core element of the Hippo/YAP axis, a highly conserved signaling pathway, and plays a vital role in tumor growth, invasion and metastasis, epithelial‐mesenchymal transformation, chemotherapy resistance, and tumor immune escape.^34^, ^35^ There is compelling evidence of the crucial role of Hippo/YAP signaling in tumor progression.^36^ For example, circRNA PPP1R12A promoted the activation of Hippo/YAP signaling to drive metastasis of colon cancer.^36^ Moreover, activated Hippo signaling was shown to be accompanied by a decrease in YAP and an increase in p‐YAP.^37^, ^38^ In the present study, WWP2 silencing was found to activate Hippo/YAP signaling, as evidenced by elevation of p‐YAP1 and downregulation of YAP1. Furthermore, LAST2 silencing‐promoted cell growth and invasion and suppressed cell ferroptosis, which was compromised by CMTM5 overexpression or WWP2 knockdown.

In conclusion, we propose that CMTM5 may regulate Hippo/YAP axis to suppress cell growth and invasion and accelerate cell ferroptosis in glioma by inhibiting WWP2‐mediated LATS2 ubiquitination. Our findings provide more targeted genes for delaying glioma progression. However, some limitations of this study should be acknowledged. For example, we did not rule out whether CMTM5 can affect other forms of tumor cell death, such as apoptosis, and autophagy. For example, CMTM5 overexpression significantly inhibited prostate cancer cell proliferation, migration and invasion, and promoted cell apoptosis compared with vector control cells in vitro.^27^ CMTM5 and CMTM7 are both members of the CMTM family, and studies have shown that both in vitro and in vivo experiments demonstrated that knockdown of CMTM7 enhanced tumor growth by impairing autophagy,^39^ indicating that CMTM5 may participate in tumor development by mediating autophagy. Further studies are required to investigate the effects of CMTM5 on other death phenotypes. Previous literature reports have shown a correlation between LATS2 or WWP2 and chemotherapy drugs, including oxaliplatin. And the study found that the transcription factor FOXC1 binding to the miR‐31 promoter increases the expression of miR‐31‐5p and regulates the expression of LATS2, leading to resistance to OXA in colon cancer cells.^37^ Overexpression of WWP2 limits the progression of multiple myeloma and enhances the sensitivity of cells to bortezomib therapy.^40^ However, it is still unclear whether the CMTM5/WWP2/LATS2 axis is involved in angiogenesis in glioma and the treatment of glioma with bevacizumab. we did not investigate whether CMTM5/WWP2/LATS2 signaling affects DNA repair enzyme O6Meg DNA methyltransferase (MGMT) status in glioma. However, no studies have reported on the relationship between CMTM5/WWP2/LATS2 signaling and MGMT, which also provides a direction for our future research. Fourthly, we did not investigate the relationship between CMTM5/WWP2/LATS2 signaling and chemotherapy temozolomide efficacy. Finally, due to time constraints, we were unable to collect enough tissue samples to detect WWP2 and LAST2 expression. Future studies should aim to overcome the limitations of this study to explore the role of CMTM5/WWP2/LATS2 signaling in glioma.

## CONFLICT OF INTEREST STATEMENT

The authors declare no conflict of interest.

## Supporting information


Figure S1
