# Supplementary material for: CMTM5 influences Hippo/YAP axis to promote ferroptosis in glioma through regulating WWP2‐mediated LATS2 ubiquitination
Source: Kaohsiung J Med Sci. 2024 Aug 21;40(10):890–902. doi: 10.1002/kjm2.12889 (PMC11895632; doi:10.1002/kjm2.12889)
Supplement: Supplementary file 1 — Figure S1 [file KJM2-40-890-s001.docx]

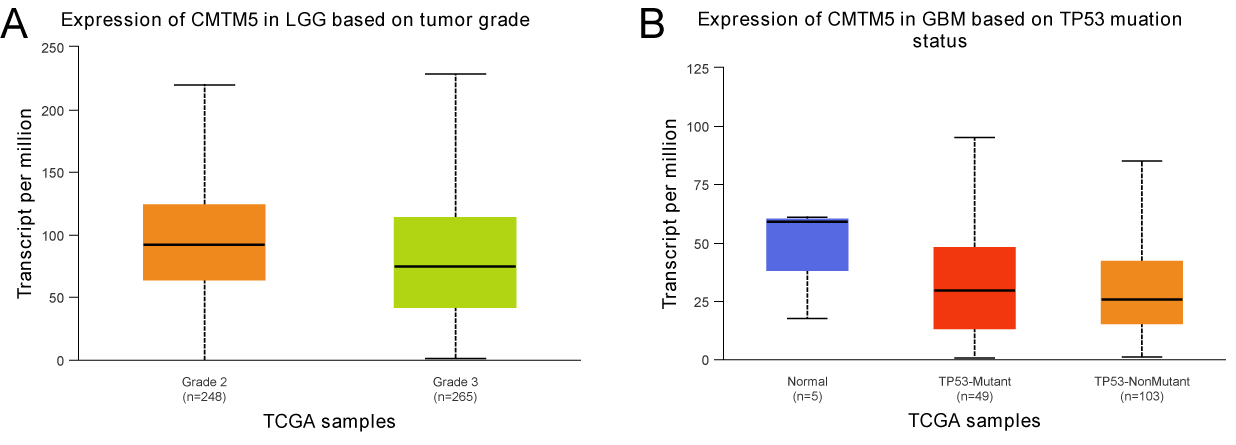


**Figure S1 The expression of CMTM5 in glioma was analyzed by CGGA database.** (A) The expression of CMTM5 in different grades glioma. (B) The expression of CMTM5 in glioma based on TP53 muation.


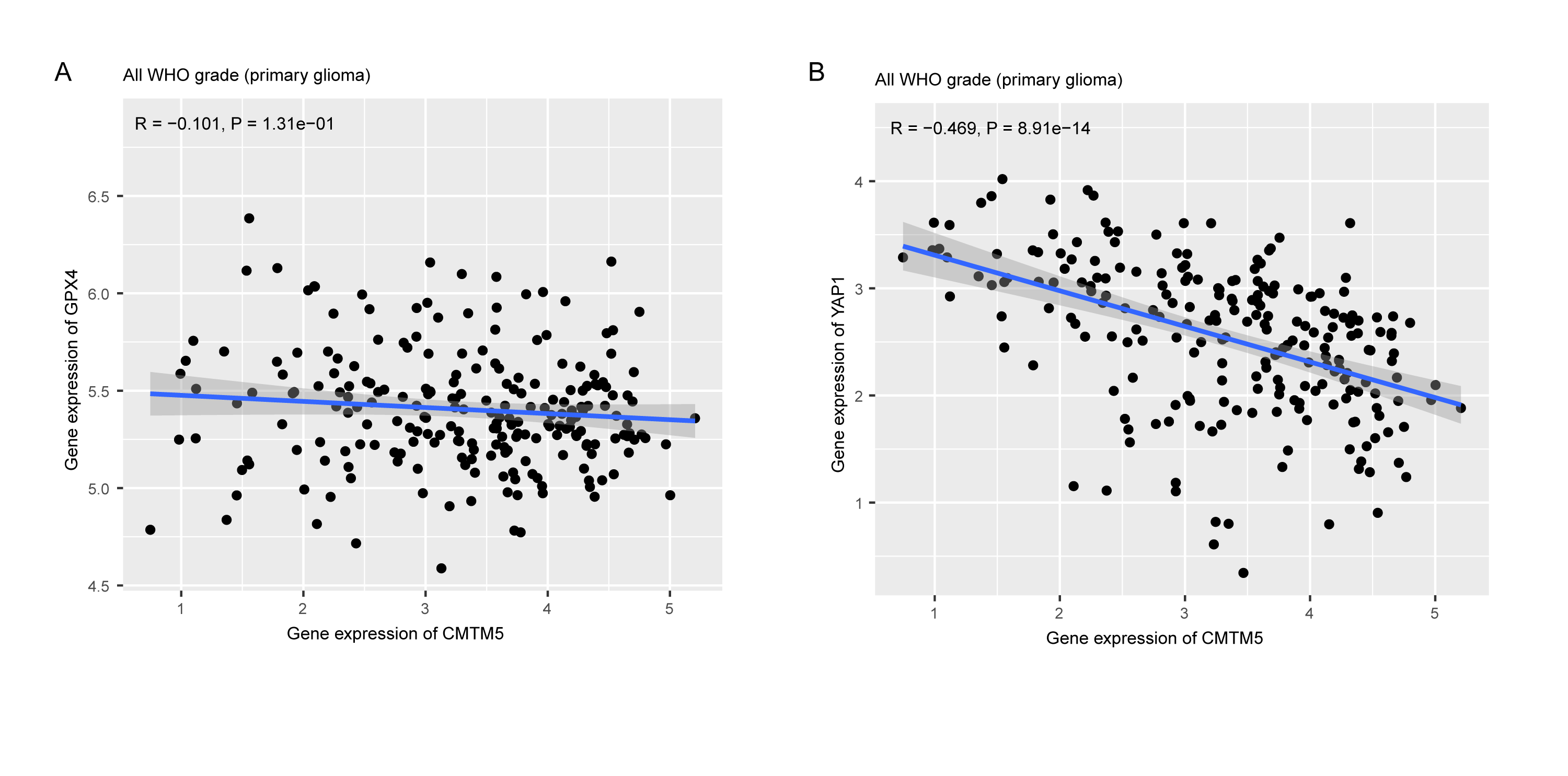


**Figure S2 The correlation between CMTM5 and different proteins was analyzed by CGGA database.** (A) Correlation analysis between CMTM5 and GPX4. (B) Correlation analysis between CMTM5 and YAP1.
